# Indole is an inter-species biofilm signal mediated by SdiA

**DOI:** 10.1186/1471-2180-7-42

**Published:** 2007-05-18

**Authors:** Jintae Lee, Arul Jayaraman, Thomas K Wood

**Affiliations:** 1 Artie McFerrin Department of Chemical Engineering, Texas A & M University, College Station, TX 77843-3122, USA; 2 Department of Biology, Texas A & M University, College Station, TX 77843-3258, USA; 3 Zachry Department of Civil Engineering, Texas A & M University, College Station, TX 77843-3136, USA

## Abstract

**Background:**

As a stationary phase signal, indole is secreted in large quantities into rich medium by *Escherichia coli *and has been shown to control several genes (e.g., *astD*, *tnaB*, *gabT*), multi-drug exporters, and the pathogenicity island of *E. coli*; however, its impact on biofilm formation has not been well-studied.

**Results:**

Through a series of global transcriptome analyses, confocal microscopy, isogenic mutants, and dual-species biofilms, we show here that indole is a non-toxic signal that controls *E. coli *biofilms by repressing motility, inducing the sensor of the quorum sensing signal autoinducer-1 (SdiA), and influencing acid resistance (e.g., *hdeABD, gadABCEX*). Isogenic mutants showed these associated proteins are directly related to biofilm formation (e.g., the *sdiA *mutation increased biofilm formation 50-fold), and SdiA-mediated transcription was shown to be influenced by indole. The reduction in motility due to indole addition results in the biofilm architecture changing from scattered towers to flat colonies. Additionally, there are 12-fold more *E. coli *cells in dual-species biofilms grown in the presence of *Pseudomonas *cells engineered to express toluene *o-*monooxygenase (TOM, which converts indole to an insoluble indigoid) than in biofilms with pseudomonads that do not express TOM due to a 22-fold reduction in extracellular indole. Also, indole stimulates biofilm formation in pseudomonads. Further evidence that the indole effects are mediated by SdiA and homoserine lactone quorum sensing is that the addition of *N*-butyryl-, *N*-hexanoyl-, and *N*-octanoyl-*L*-homoserine lactones repress *E. coli *biofilm formation in the wild-type strain but not with the *sdiA *mutant.

**Conclusion:**

Indole is an interspecies signal that decreases *E. coli *biofilms through SdiA and increases those of pseudomonads. Indole may be manipulated to control biofilm formation by oxygenases of bacteria that do not synthesize it in a dual-species biofilm. Furthermore, *E. coli *changes its biofilm in response to signals it cannot synthesize (homoserine lactones), and pseudomonads respond to signals they do not synthesize (indole).

## Background

It has been established that cell-to-cell signaling plays a role in the formation of some biofilms. For example, cell signaling controls the production and secretion of exopolysaccharides for *Vibrio cholerae *biofilms [1]. This signaling may be complex as *V. harveyi *uses three cell-sensing signals including *N*-(3-hydroxybutanoyl) homoserine lactone (autoinducer-1, AI-1), furanosyl borate diester (AI-2), and a signal, synthesized by CqsA, whose structure is unknown [2]. In addition, the quorum sensing signal *N*-(3-oxododecanoyl)-*L*-homoserine lactone controls biofilm formation in *Pseudomonas aeruginosa *[3], and we have found that in vitro synthesized AI-2 stimulates directly *Escherichia coli *biofilm formation [4,5].

Indole is an extracellular signal in *E. coli *as it has been shown to regulate expression of *astD*, *tnaB*, and *gabT *in the stationary phase for planktonic cells [6]. Indole has also been shown to control multi-drug exporters in *E. coli *[7] as well as to regulate the pathogenicity island of pathogenic *E. coli *[8] (note tryptophanase activity has also been linked to killing of nematodes by *E. coli *but indole is not directly responsible for this effect [8]). Recently, indole has been shown to link plasmid multimerization and cell division [9]. Using DNA microarrays, we discovered that genes for the synthesis of indole (*tnaAL*) were induced by a stationary phase signal [10] and that the gene encoding tryptophanase, *tnaA*, was repressed 13-fold in 6-day-old *E. coli *biofilms in complex medium [11]. These results implied that indole plays a role in biofilm formation since biofilm cells most closely resemble stationary-phase cells [12,13]. Using two *E. coli *mutants *yliH *and *yceP*, we found that indole probably inhibits biofilm formation since these two mutations lead to biofilms with lower intracellular indole concentrations which leads to dramatic increases in biofilm formation and since the addition of extracellular indole reduced biofilm formation for these mutants [14]. In contrast, others have reported that indole induces biofilm formation in *E. coli *as the *tnaA *deletion decreased biofilm formation and the addition of indole restored it [15]. Hence, we sought here to explore this contradiction using DNA microarrays so that we could study the whole genome as well as use isogenic mutants to test our hypotheses. This approach has led to both the discovery and elucidation of the role of the biofilm regulators MqsR [4], BssR/BssS [14], Hha/YbaJ [16], and TqsA [5].

The physiological role of SdiA has been unclear in *E. coli *[17]. SdiA is a LuxR homologue that is a quorum-sensing-regulated transcription factor in *E. coli *[18]*; *in other bacteria, LuxR systems control density-dependent gene regulation through homoserine lactones but *E. coli *does not have a homoserine lactone synthase [19]. In *E. coli *O157:H7, SdiA has been shown to regulate virulence factors [20], and SdiA has been shown (by overexpressing SdiA from a plasmid) [21] to inhibit chemotaxis and motility genes in *E. coli *K-12, to repress *tnaA*, as well as to induce indole export via AcrEF [6]. Recently, it has been determined that SdiA responds to three different homoserine lactone signals [19,22], and that SdiA controls acid resistance via a synthetic homoserine lactone [23].

**Figure 1:**
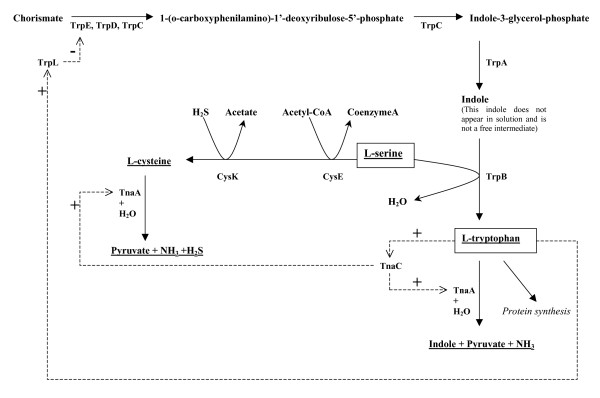
Major components of the tryptophan pathway. Dashed lines indicate regulation.

To investigate the role of indole in biofilms, the isogenic mutations *tnaA *(encoding tryptophanase), *trpE *(encoding anthranilate synthase component I), *tnaC *(encoding the tryptophanase leader peptide), and *trpL *(encoding Trp operon leader peptide) were used since they control indole synthesis in *E. coli *[24] (Fig. 1). It was found here that homoserine lactone quorum sensing is related to *E. coli *biofilms via SdiA, and that indole is an inter-species biofilm signal that may be manipulated by other bacteria. In addition, the regulation of biofilms via indole is linked to acid resistance via known paths (e.g., *hdeABD, gadABCEX*). It was also found that homoserine lactone signals repress *E. coli *biofilm formation.

## Results

The goals of this research were to determine whether indole influences *E. coli *and *Pseudomonas *sp. biofilm formation (using crystal violet staining for rapid results and flow cells to examine biofilm architecture) and to determine the genetic basis of this influence through DNA microarrays and isogenic mutants. The concentration of indole used here (~500 μM) was not toxic to *E. coli *since the growth rate at 500 μM was reduced only by 7.6%.

### Tryptophan mutations and indole concentrations

**Figure 2:**
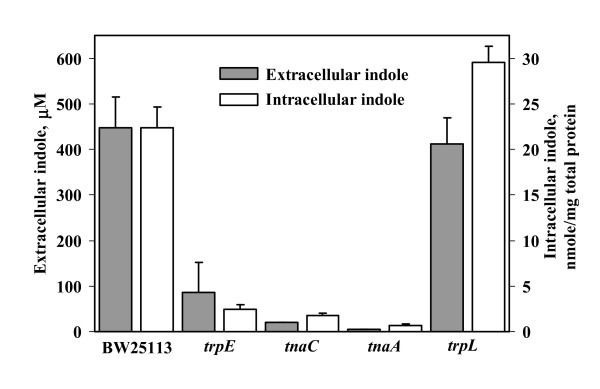
Intracellular and extracellular indole concentration in LB for BW25113, BW25113 *trpE*, BW25113 *tnaC*, BW25113 *tnaA*, and BW25113 *trpL*. Each experiment was performed in duplicate, and one standard deviation is shown.

The *trpE, tnaC*, and *tnaA *mutations in the tryptophan pathway (Fig. 1) reduced by a factor of 10 the intracellular indole concentration for *E. coli *K-12 in Luria-Bertani medium (LB) (Fig. 2). The *trpL *strain had 30% more intracellular indole compared to the wild-type strain (Fig. 2) since *trpL *encodes the attenuator (Fig. 1). In addition, extracellular indole concentrations were reduced for the *trpE, tnaC*, and *tnaA *mutations (Fig. 2, 450 μM reduced to 5 to 90 μM).

### Biofilm formation

**Figure 3:**
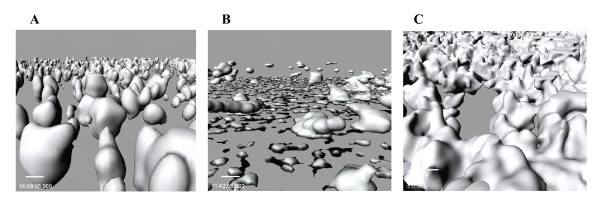
Biofilm formation in LB glu at 24 h in flow cells (A) with wild-type K-12 BW25113, (B) with wild-type K-12 BW25113 with 500 μM indole, and (C) with K-12 BW25113 *trpE*. Scale bar is 5 μm.

Since we have measured extracellular indole concentrations greater than 600 μM with wild-type K12 [14], we added 500 μM indole to *E. coli *K-12 in LB supplemented with 0.2 % (w/v) glucose (LB glu) and found it decreases biofilm formation in flow cells (Fig. 3). LB glu was chosen since it reduces background indole concentrations (due to catabolite repression of *tnaA *[25]) so that exogenous indole would have a greater effect. The addition of indole to the wild-type *E. coli *changed the biofilm architecture from scattered towers to flatter colonies (Fig. 3A/3B). COMSTAT analysis (Table 1) indicated biomass was reduced 40% and substratum coverage was increased 2.8-fold due to the flat nature of the biofilms with indole.

**Table 1:** Biofilm COMSTAT flow cell measurements for *E. coli *BW25113 and its isogenic mutants LB glu medium at 24 h and for the dual species (*E. coli/P. fluorescens*) biofilm in LB medium after 5 days

**Conditions**	**Strains**	**Biomass, μm^3^/μm^2^**	**Substratum coverage, %**	**Mean thickness, μm**	**Roughness coefficient**
Single species of BW25113 and its isogenic mutant	BW25113	9 ± 5	8 ± 9	24 ± 8	0.6 ± 0.4
	BW25113 with DMF (control for indole)	6.4 ± 3	6.5 ± 7	21 ± 8	0.5 ± 0.5
	BW25113 with 500 μM indole	4 ± 1.5	18 ± 6	25 ± 6	0.9 ± 0.6
	BW25113 *trpE*	30 ± 7	13 ± 6	46 ± 3.5	0.3 ± 0.1

Dual species with TOM	*P. fluorescens *2-79TOM/pHKT3	0.06 ± 0.05	0.90 ± 0.56	0.14 ± 0.13	1.97 ± 0.02
	*E. coli *K-12 XL1-Blue/pCM18	6.63 ± 2.67	21.52 ± 10.49	13.95 ± 5.67	0.90 ± 0.34

Dual species without TOM	*P. fluorescens *2-79/pHKT3	0.03 ± 0.01	0.64 ± 0.16	0.04 ± 0.02	1.99 ± 0.01
	*E. coli *K-12 XL1-Blue/pCM18	0.56 ± 0.51	1.81 ± 1.23	1.04 ± 0.68	1.87 ± 0.06

In 96 wells using crystal violet, addition of 1000 μM indole in LB at 30°C also decreased the 8 h biofilm of *E. coli *ATCC25404 by 46 ± 22%, of JM109 by 13 ± 8%, of TG1 by 77 ± 13%, and of XL1-Blue by 44 ± 7%. Hence, indole decreased *E. coli *biofilm formation.

**Figure 4:**
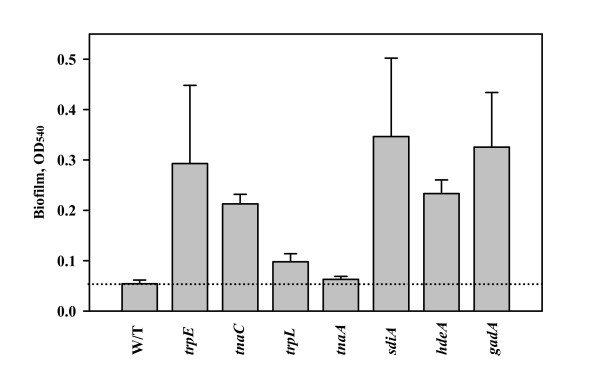
Effect of the *trpE*, *tnaC*, *trpL*, *tnaA, sdiA, hdeA*, and *gadA *mutations on biofilm formation in LB glu media. Biomass measured at 540 nm after 24 h. Each experiment was repeated two or four times, and one standard deviation is shown.

We also explored biofilm formation with several tryptophan pathway mutants using the crystal violet stain method. Along with the *trpE, tnaC*, and *trpL *strains, a strain with the isogenic mutation *tnaA *was also studied for its effect on biofilm formation because these genes are required for the synthesis of indole [24] (Fig. 1). Deletion of *trpE *and *tnaC *(both decrease indole) increased biofilm formation in LB glu medium (Fig. 4) (5.4- and 3.9-fold at 24 h, respectively). As expected, addition of 500 and 1000 μM indole to these two mutants reduced biofilm formation in a dose-dependent response to wild-type levels (2.1- and 4.4-fold reduction for *trpE *and 1.3- and 3.5-fold for *tnaC*, respectively). These results show reducing intracellular indole concentrations increases biofilm formation in *E. coli*. They also corroborate our earlier results in which the addition of 250 μM indole reduced biofilm formation in the isogenic *yceP *and *yliH *mutants in LB and LB glu; these two strains overproduce biofilm as a result of reduced intracellular indole concentrations [14]. The deletion of *tnaA *did not affect biofilm formation in LB glu medium (Fig. 4) since *tnaA *is under catabolite repression.

To verify the 96-well biofilm assay, biofilm formation in LB glu was tested in a continuous flow system for the *trpE *mutant (rather than the repressed *tnaA *mutant), and the changes in biofilm were quantified using COMSTAT [26]. The *trpE *mutant displayed similar results in the flow cell as compared to the 96 wells as its deletion increased biomass (3.3-fold) and thickness (2-fold) (Fig. 3A/3C, Table 1).

### Dual-species biofilms

Toluene *o-*monooxygenase (TOM) of the soil bacterium *Burkholderia cepacia *G4 converts indole into isoindigo [27]; hence, we hypothesized that if a bacterium other than *E. coli *degraded indole and if indole represses *E. coli *biofilm formation, then *E. coli *would be present in higher numbers in a biofilm that had another bacterium which expressed TOM. TOM was integrated into the chromosome of the *P. fluorescens *strain to diminish the metabolic burden of this locus [28] and because this strain does not produce indole. At day 7 in the flow cell with LB medium, the dual species with the *Pseudomonas *expressing TOM showed 2- to 5-fold more biofilm of red fluorescent protein (RFP) expressing *E. coli *K-12 ATCC25404/pGEM-T RFP than that in dual species without TOM in duplicate experiments (data not shown). Thus, including an organism that actively expressed TOM increased the amount of *E. coli *biofilm.

**Figure 5:**
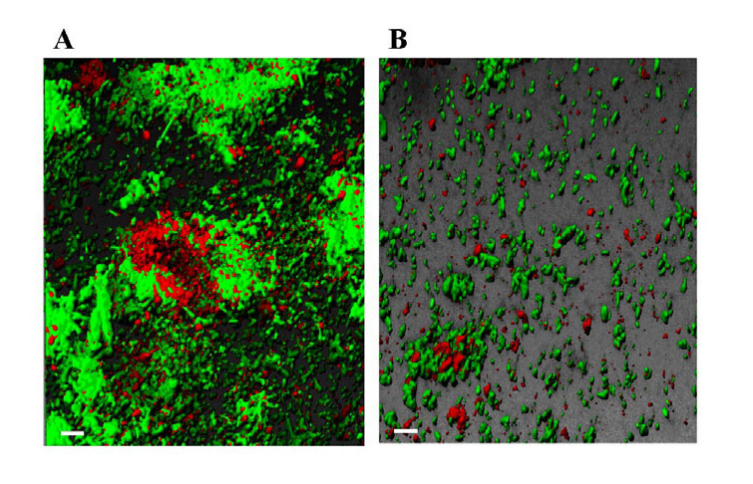
Biofilm formation in LB after 5 days in flow cells for (A) dual species of *E. coli *K-12 XL1-Blue/pCM18 (green due to GFP) and *P. fluorescens *2-79TOM/pHKT3 expressing TOM (red due to RFP), and (B) dual species of *E. coli *K-12 XL1-Blue/pCM18 (green due to GFP) and *P. fluorescens *2-79/pHKT3 (red due to RFP). Scale bar is 10 μm.

Similarly, when the *Pseudomonas *was tagged with RFP and *E. coli *with green fluorescent protein (GFP) so that both bacteria could be visualized, constitutive expression of TOM from the chromosome led to a 12-fold increase in *E. coli *biofilm after five days in the flow cell (Fig. 5 and Table 1). By expressing TOM to remove indole, both substratum coverage and mean thickness increased 10-fold; hence, sparse microcolonies became more mature colonies in the absence of the biofilm inhibitor indole. These corroborating sets of dual species biofilm results support our hypothesis that indole decreases biofilm formation with *E. coli*. Since a mixed biofilm formation of two species of *Pseudomonas *and *E. coli *was clearly observed (Fig. 5), this experiment also demonstrates that other bacteria may interfere with indole to control the biofilm formation of *E. coli*.

To ensure that extracellular indole is present, we ran the flow cell with wild-type K-12 alone and found that indole concentrations increase from 17 to 250 μM in 2 to 19 h then steadily decrease to 156 μM in 43 h, so substantial indole is present due to *E. coli *in our flow cells. Then, to show directly that the TOM-expressing pseudomonad decreased the indole levels, we measured extracellular indole at 8 h in LB medium (via high-pressure liquid chromatography, HPLC) for the RFP/GFP dual-culture system with TOM vs. no TOM and found that the indole concentration was decreased 22-fold (240 ± 16 μM for *E. coli *XL1-Blue/pCM18/*P. fluorescens *2–79/pHKT3 that lacks TOM vs. 11 ± 3 μM for *E. coli *XL1-Blue/pCM18/*P. fluorescens *2-79TOM/pHKT3 expressing TOM). The cell growth rate was 0.45 ± 0.02/h for *P. fluorescens *2-79/pHKT3 and 0.52 ± 0.02/h for *P. fluorescens *2-79TOM/pHKT3; hence, the extracellular indole concentrations were decreased by cloning TOM, and the changes in *E. coli *biofilm formation are due to a reduction in extracellular indole concentrations.

### Indole and pseudomonad biofilms

To determine if indole is a signal for pseudomonads, too, we added indole to *P. aeruginosa*. Indole at 500 μM increased biofilm formation 1.4-fold and 1000 μM increased biofilm formation 2.2-fold (crystal violet density 1.0 ± 0.2 vs. 2.2 ± 0.2); hence, indole, although not synthesized by *P. aeruginosa *(extracellular concentration was 0 μM), is a signal that stimulates biofilm in this strain. In addition, removing indole (1000 μM) by expressing TOM in *P. fluorescens *2–79 results in a 5.6-fold reduction in *P. fluorescens *biomass compared to *P. fluorescens *2–79 without TOM which indicates that indole stimulates biofilm formation in this pseudomonad (crystal violet density 0.45 ± 0.07 vs. 0.08 ± 0.01).

### External indole decreases motility and the deletion of *trpE *or *tnaC *increases motility

**Figure 6:**
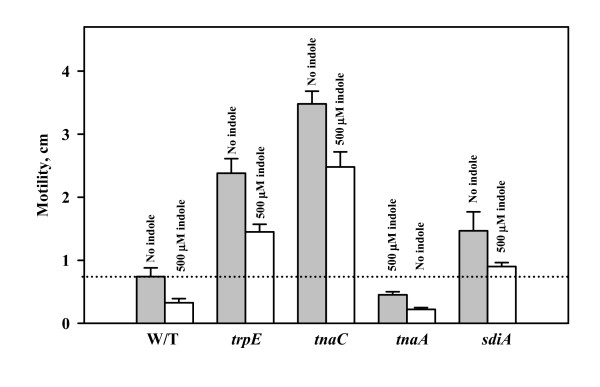
Effect of indole (500 μM) on the motility of BW25113 wild-type (W/T), BW25113 *trpE*, BW25113 *tnaC*, BW25113 *tnaA*, and BW25113 *sdiA*. Motility halos were measured at 8 h. Each experiment was repeated two or four times, and one standard deviation is shown. DMF (0.1 %, v/v) was used as a negative control.

To investigate the cause of the reduction in biofilm due to indole, motility was studied since it positively influences biofilm formation in *E. coli *[29,30]. The deletion of *trpE *and *tnaC *increased motility (3.2-fold for *trpE *and 4.7-fold for *tnaC*) compared with the isogenic wild-type strain (Fig. 6). Therefore the reduction in intracellular indole concentration through the deletion of *trpE *and *tnaC *increases motility which leads to the increased biofilm formation; hence, indole decreases motility. To corroborate that indole decreases motility, the motility of wild-type *E. coli *and its *trpE *and *tnaC *mutants were tested upon addition of 500 μM indole; the addition of indole decreased motility by 30 to 40% for these strains (Fig. 6), so the enhanced motility due to a reduction of indole could be diminished by direct addition of indole. Hence, indole reduces motility in *E. coli*, and this reduced motility appears be one of causes of the reduced biofilm formation.

### Indole is a global regulator in biofilm formation in *E. coli *through SdiA and acid resistance

To investigate the genetic basis of indole regulation of biofilms, we performed three sets of microarray experiments: (i) direct addition of 600 μM indole to K-12 *yceP *in LB glu since this strain has elevated biofilm formation due to low intracellular indole and the biofilm responds to added indole [14] (note two independent sets of microarrays were used for this experiment), (ii) a comparison of K-12 *trpE *vs. K-12 wild-type in LB glu since the *trpE *cells had reduced indole and had elevated biofilm in LB glu at 24 h, and (iii) K-12 *tnaA *vs. K-12 wild-type in LB since the *tnaA *cells also had little detectable indole in this medium. Glass wool was used to increase the surface area so that RNA could be readily obtained for the microarrays. The most significantly induced and repressed genes are shown in Table 2. Thirteen, 43, and 34 genes were differentially induced (greater than 2 fold) and 17, 21, and 141 genes were differentially repressed (greater than 2 fold) upon indole addition, deleting *trpE*, or deleting *tnaA*, respectively.

**Table 2:** Partial list of genes induced and repressed more than 2-fold after 24 h in (i) K-12 *yceP *biofilms upon addition of 600 μM indole in LB glu medium (experiment performed in duplicate, and one standard deviation is shown), (ii) K-12 biofilms due to the *trpE *mutation in LB glu medium, and (iii) K-12 biofilms due to the *tnaA *mutation in LB medium.

**Gene**	**b #**	**Fold change upon indole addition**	**Fold change upon *trpE *deletion**	**Fold change upon *tnaA *deletion**	**Description**	**Protein Size, aa**
**Transcription**						
*sdiA*	*b*1916	**2.9 ± 0.8**	1.0*	1.2	AHL transcriptional activator (LuxR/TraR family)	240
*ygaV*	*b*2667	**2.2 ± 0.2**	1.1*	-1.1*	hypothetical protein	99
*soxS*	*b*4062	**2.3 ± 0.3**	**-2.8**	-1.2	regulation of superoxide response regulon	107
*cspA*	*b*3556	1.8 ± 0.4	**-4.6**	-1.2*	cold shock protein 7.4, transcriptional activator of hns	70
*mtlR*	*b*3601	1.3* ± 0.4	**-2.6**	-1.4*	MtlR transcriptional repressor	195
*yjcT*	*b*4084	-1.1* ± 0.1	1.5*	**-3.5**	D-allose kinase	309
*rhoL*	*b*3782	**2.2 ± 0.1**	-1.1*	1.1*	rho operon leader peptide	33
**Cell motility and secretion**						
*hha*	*b*0460	**4.9 ± 0.0**	-1.4	-1.4	haemolysin expression modulating protein	72
*ybaJ*	*b*0461	**5.2 ± 0.8**	-1.1*	1.0*	conserved hypothetical protein	124
*mqsR*	*b*3022	**2.8 ± 0.7**	1.1*	1.4	master regulator of chemotaxis via AI-2; interacts with QseB	98
*sfmA*	*b*0530	-1.3* ± 0.1	1.0*	**-2.3**	putative fimbrial-like protein	191
*sfmC*	*b*0531	-1.1* ± 0.1	-1.1*	**-2.3**	putative chaperone	230
*sfmH*	*b*0533	-1.2* ± 0.1	1.1*	**-2.0**	fimbrial assembly protein	325
*flgA*	*b*1072	1.1* ± 0.1	1.2*	**-2.1**	flagellar biosynthesis; assembly of basal-body periplasmic P ring	219
*yehA*	*b*2108	1.1* ± 0.0	-1.1*	**-2.1**	hypothetical protein	344
*yhcA*	*b*3215	1.1* ± 0.0	-1.7	**-2.0**	putative chaperone	224
*yfcP*	*b*2333	1.1* ± 0.1	1.3*	**-2.0**	putative fimbrial-like protein	179
**Colanic acid synthesis genes**						
*ypdI*	*b*2376	-1.2* ± 0.2	1.3*	**-2.5**	YpdI colanic acid synthesis lipoprotein	91
**Acid resistance**						
*gadE*	*b*3512	**-4.3 ± 1.4**	-1.6	1.1*	activator of acid resistance genes and putative LuxR transcriptional activator	175
*gadA*	*b*3517	**-4.0 ± 1.3**	**-2.3**	1.2*	glutamate decarboxylase A, isozyme, PLP-dependent	466
*gadB*	*b*1493	**-2.8 ± 0.7**	**-2.6**	1.2*	glutamate decarboxylase isozyme	466
*gadC*	*b*1492	**-3.7 ± 0.0**	-1.6	1.2*	acid sensitivity protein, putative transporter, encoding a γ-aminobutyrate antiporter	511
*gadX*	*b*3516	**-2.0 ± 0.4**	-1.2	-1.2	activator of *gadA *and *gadBC*	274
*hdeA*	*b*3510	**-4.8 ± 0.2**	**-2.3**	1.0*	periplasmic chaperone of acid-denatured protein	110
*hdeB*	*b*3509	**-3.9 ± 0.6**	**-2.3**	1.2*	10K-L protein, periplasmic protein related to acid resistance protein	112
*hdeD*	*b*3511	**-3.0 ± 0.5**	-1.6	-1.5	protein involved in acid resistance	190
**Phage-related genes**						
*cspI*	*b*1552	1.8 ± 0.7	1.0*	**-3.7**	Qin prophage; cold shock-like protein	70
*ypjF*	*b*2646	1.4* ± 0.2	1.2*	**-12.1**	CP4-57 prophage	109
*ymfI*	*b*1143	1.1* ± 0.1	1.1*	**-5.7**	E14 prophage	113
*ydaY*	*b*1366	1.0* ± 0.0	1.0*	**-4.6**	Rac prophage	119
*ydfP*	*b*1553	-1.2* ± 0.2	1.2*	**-2.6**	Qin prophage	165
*ydfE*	*b*1577	1.0* ± 0.1	1.3*	**-2.5**	Qin prophage	255
*yeeV*	*b*2005	-1.5* ± 0.5	1.1*	**-2.5**	CP4-44 prophage	124
*stfE*	*b*1157	1.4* ± 0.4	-1.1*	**-2.3**	E14 prophage; putative tail fiber protein	166
*b*1364	*b*1364	1.2* ± 0.1	1.1*	**-2.3**	Rac prophage	93
*yfjW*	*b*2642	1.4* ± 0.4	-1.1*	**-2.1**	CP4-57 prophage	567
*yeeU*	*b*2004	1.0* ± 0.0	-1.1*	**-2.0**	CP4-44 prophage; putative structural protein	122
*yfjT*	*b*2637	-1.4* ± 0.6	1.1*	**-2.0**	CP4-57 prophage	155
*ynaE*	*b*1375	1.8 ± 0.6	-1.9	**-2.0**	Rac prophage	88
**Amino acid transport and metabolism**						
*tnaA*	*b*3708	1.7 ± 0.0	-1.3*	**-14.9**	tryptophan deaminase, PLP-dependent	476
*tnaC*	*b*3707	1.5 ± 1.1	1.1*	**32.0**	tryptophanase leader peptide	24
*mtr*	*b*3161	-1.1* ± 0.1	-1.3	**-5.7**	Mtr tryptophan ArAAP transporter	414
*aroP*	*b*0112	1.0* ± 0.0	-1.2*	**-5.3**	AroP phenylalanine/tyrosine/tryptophan APC transporter	457
*proC*	*b*0386	-1.2 ± 0.0	1.0*	**-3.0**	pyrroline-5-carboxylate-reductase	269
**Carbohydrate transport and metabolism**						
*melB*	*b*4120	1.2* ± 0.1	1.1*	**-7.5**	melibiose permease II	469
*eno*	*b*2779	**-1.9 ± 0.2**	-1.2*	1.1*	Enolase	432
*yegB*	*b*2077	-1.2* ± 0.2	1.1*	**-3.2**	multidrug transport protein (MFS family)	
*prpB*	*b*0331	**-2.4 ± 0.6**	-1.1*	1.0*	putative carboxyphosphonoenolpyruvate mutase	296
**Other metabolism**						
*htrL*	*b*3618	**2.2 ± 0.1**	1.1*	-1.2*	involved in lipopolysaccharide biosynthesis	290
*pyrG*	*b*2780	**-2.2 ± 0.2**	1.0*	1.0*	CTP synthetase	545
*pheL*	*b*2598	-1.2 ± 0.1	**-4.9**	**2.3**	chorismate mutase-P-prephenate dehydratase leader peptide	15
*yodA*	*b*1973	1.5 ± 0.4	1.0*	**-7.5**	cadmium-induced metal binding protein	216
*chaA*	*b*1216	-1.4* ± 0.4	1.2*	**-7.0**	sodium-calcium/proton antiporter	366
*nhaA*	*b*0019	-1.2* ± 0.0	-1.4	**-3.2**	Na+/H antiporter, pH dependent	388
*ybdS*	*b*0612	-1.3* ± 0.3	-1.2*	**-3.2**	citrate carrier	487
**Energy production and conversion**						
*rsxA*	*b*1627	1.1* ± 0.0	-1.1*	**-14.9**	integral membrane protein of SoxR-reducing complex	193
*ynbA*	*b*1408	1.1* ± 0.0	1.4*	**-4.6**	putative diacylglycerol cholinephosphotransferase	203
**Posttranslational modification, protein turnover, chaperones**						
*htpX*	*b*1829	**2.8 ± 1.1**	-1.1*	1.1*	heat shock protein, integral membrane protein	293
**Translation**						
*ksgA*	*b*0051	-1.9 ± 0.6	-1.3*	**-3.0**	S-adenosylmethionine-6-N',N'-adenosyl (rRNA) dimethyltransferase	273
*infA*	*b*0884	1.0* ± 0.1	-1.3*	**-3.0**	protein chain initiation factor IF-1	72
**Defense mechanisms**						
*soda*	*b*3908	1.2* ± 0.3	**-3.2**	1.1*	superoxide dismutase, manganese	206
**Poorly-characterized genes**						
*ycfR*	*b*1112	**2.7 ± 0.8**	1.0*	-1.4	hypothetical protein	85
*ylaD*	*b*0459	**2.3 ± 0.3**	1.0*	-2.8*	maltose O-acetyltransferase	183
*yebE*	*b*1846	**2.4 ± 0.1**	1.1*	-2.1	hypothetical protein	219
*yncJ*	*b*1436	**2.7 ± 0.5**	-1.1*	-1.2	hypothetical protein	76
*yejG*	*b*2181	**2.4 ± 0.4**	-1.5	-1.2*	hypothetical protein	114
*ybjM*	*b*0848	-1.3* ± 0.3	-1.4	**-8.6**	hypothetical protein	125
*b*0309	*b*0309	-1.5* ± 0.6	-1.4	**-4.6**	hypothetical protein	70
*ypjB*	*b*2649	1.1* ± 0.0	1.2*	**-3.7**	hypothetical protein	263
*apaG*	*b*0050	**-2.2 ± 0.9**	-1.3	**-3.7**	hypothetical protein	125
*ydiY*	*b*1722	-1.1* ± 0.1	1.2*	**-3.5**	hypothetical protein	252
*ymfA*	*b*1122	-1.1* ± 0.1	1.0*	**-3.5**	hypothetical protein	156
*yeiU*	*b*2174	1.2* ± 0.1	-1.2*	**-3.0**	hypothetical protein	249
*yahO*	*b*0329	**-1.8 ± 0.4**	1.3	1.3	hypothetical protein	91
*psiF*	*b*0384	**-1.9 ± 0.3**	-1.1*	-1.3	induced by phosphate starvation; pho regulon member, requiring phoRB system	112
*ycdF*	*b*1005	**-2.0 ± 0.1**	1.1*	-1.2	hypothetical protein	76
*yciG*	*b*1259	**-2.1 ± 0.1**	1.2*	1.6	hypothetical protein	78
*ycgZ*	*b*1164	**-2.9 ± 0.6**	1.3	1.7	hypothetical protein	78
*ymgC*	*b*1167	**-2.1 ± 0.3**	1.2*	1.5	hypothetical protein	82
*ymgA*	*b*1165	**-2.4 ± 1.3**	1.1	1.7	hypothetical protein	90
*ymgB*	*b*1166	**-5.2 ± 1.3**	1.2	1.5	hypothetical protein, putative acid-resistance protein	88

Full data available using GEO accession number 4562. Asterisk indicates p value greater than 0.05 (data are less reliable but included for completeness). Negative values indicate repressed genes. b # indicates the Blattner number for each gene. Boldface indicates most significant fold changes.

Notably SdiA was one of the most-induced genes (2.9-fold) upon addition of 600 μM indole (Table 2). Since SdiA is predicted to inhibit chemotaxis and motility based on microarrays [21], it was expected that a *sdiA *deletion should lead to enhanced motility and biofilm formation. Corroborating our microarray results, the motility of the isogenic *sdiA *strain was increased 2.0 ± 0.6-fold, and biofilm formation was increased 6 ± 2-fold in LB glu at 37°C at 24 h (Fig. 4) and increased 3.5-fold in LB at 30°C at 24 h. In addition, for short time experiments (8 h), the *sdiA *mutation caused a 51-fold increase biofilm formation at 30°C in LB (absorbance at 540 nm of 1.53 ± 0.07 vs. 0.03 ± 0.01); however, there was no change in biofilm formation upon deleting *sdiA *at 37°C with LB. Hence, SdiA represses motility and biofilm formation.

As expected, the addition of 1000 μM indole to the *sdiA *mutant in LB glu did not appreciably decrease its elevated biofilm levels (data not shown). Hence, indole induces expression of SdiA which appears to result in SdiA repressing biofilm formation by decreasing motility. Similarly, the *trpE *mutation, which diminishes intracellular indole, led to both increased biofilm (Fig. 3C) and motility (Fig. 6) indicating again that indole is a biofilm inhibitor that controls biofilms by reducing motility. However, the addition of indole also decreased motility of the *sdiA *mutant (Fig. 6) so that other factors are involved in the motility reduction with indole. Recently, it has been reported that indole is responsible for delaying cell division, and addition of 4 mM indole stopped *E. coli *cell division [9]. Therefore, we propose that indole may decrease motility through cell division interference.

### Indole controls *sdiA*-mediated transcription

To corroborate that indole influences SdiA and the genes it controls, we tested the ability of indole to alter SdiA-influenced transcription of the *ftsQ2p *(or *ftsQp*_2_) promoter; this promoter is one of the few promoters SdiA is known to directly induce [19,31]. The addition of 1000 μM indole to *E. coli *UT481/pCX39 led to a 33 ± 15% (average of five independent cultures) decrease in *ftsQ2p *expression which agrees with the 30% reduction seen by García-Lara et al. [31] due to the unknown stationary-phase factor. Therefore, indole affects SdiA-mediated transcription. As expected, indole addition to the isogenic *sdiA *mutant (*E. coli *WX2/pCX39) had no effect on *ftsQ2p *expression (the absolute level of β-galactosidase was also 48% lower in this mutant); hence, indole either binds SdiA directly or through some intermediate and regulates SdiA-mediated transcription in the absence of acyl-homoserine lactones.

### Homoserine lactones and *E. coli *biofilm formation

Since SdiA binds four different homoserine lactone signals that *E. coli *cannot synthesize [22,23], we tested the ability of homoserine lactone signals to control biofilm formation to cement the link between SdiA and biofilms. As with indole, adding three naturally-occurring homoserine lactone signals (*N*-butyryl-*DL*-homoserine lactone [32], *N*-hexanoyl-*DL*-homoserine lactone [33], and *N*-octanoyl-*DL*-homoserine lactone [33]) inhibited K-12 biofilm formation in LB medium in a dose-dependent manner without inhibiting growth by 25%, 27%, and 18%, respectively; however, the isogenic *sdiA *mutant does not respond to the homoserine lactone signals. *N*-(3-oxooctaneoyl)-*DL*-homoserine lactone, *N*-decanoyl-*DL*-homoserine lactone, and *N*-dodecanoyl-*DL*-homoserine lactone did not change biofilm formation of either the wild type and *sdiA *mutant. These results were repeated 5 times for *N*-butyryl-*DL*-homoserine lactone and each time there was consistent and significant reduction in *E. coli *biofilm formation as long as SdiA was present. Also, the addition of 10 μM *N*-butyryl-*DL*-homoserine lactone caused a 40 ± 7% increase in *ftsQ2p *expression which confirms the homoserine lactone signal binds SdiA, while the addition of the 10 μM *N*-butyryl-*DL*-homoserine lactone to the isogenic *sdiA *mutant had no effect on *ftsQ2p *expression. Hence, *E. coli *responds to homoserine lactone signals by altering its biofilm formation and it does so through SdiA.

### Indole and acid resistance

**Figure 7:**
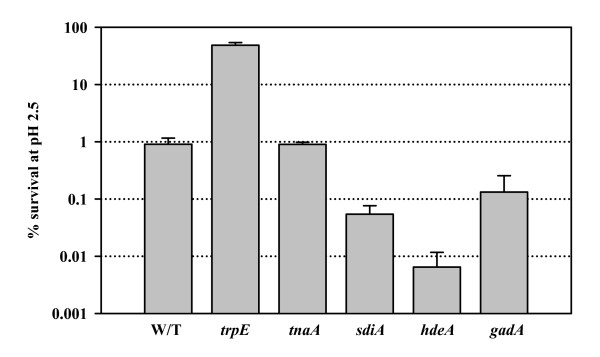
Acid resistance of BW25113 wild-type (W/T) and various knockout mutants in LB medium (pH 2.5) at 37°C. Each experiment was repeated two or four times and one standard deviation is shown.

Indole addition also repressed the glutamate decarboxylase acid-resistance genes *gadABCEX *2- to 4-fold (Table 2). GadABC are regulated by GadE and protect *E. coli *at pH 2 and below which allows the bacterium to colonize the gastrointestinal tract [34]. Also, the other known acid-resistance genes and *hdeABD *(which function as chaperones to prevent aggregation of periplasmic proteins under extremely acidic conditions [35]) were repressed 3- to 5-fold by indole (Table 2). Hence, we surmised that indole decreases acid resistance. This hypothesis was tested using LB medium at pH 2.5 and found that the *trpE *mutant (which produces 10 times less indole, Fig. 2) was 53 times less sensitive to pH 2.5 than the wild-type, while *sdiA *mutant showed 17-fold less survival (Fig. 7). As expected, the positive controls, cells with *hdeA *and *gadA *mutations displayed increased acid sensitivity (Fig. 7). To show clearly that indole is directly related to acid resistance, we investigated whether indole addition affects *E. coli *K-12 survival at pH 2.5. Addition of 2 mM indole to the wild-type strain decreased survival by 350 to 650-fold. However, addition of 2 mM indole to the *sdiA *mutant did not appreciably change acid survival (3.9-fold decrease for *sdiA *mutant). The results also support our hypothesis that indole controls biofilm formation and acid resistance via SdiA.

### *tnaA *and *trpE *microarrays

The deletion of *tnaA *led to more extensive differential gene expression compared to the deletion of *trpE *and indole addition to the *yceP *mutant. As expected, *tnaC *was highly induced (32-fold, Table 2) indicating high concentrations of tryptophan arise when TnaA is not active. *mtr*, which encodes the tryptophan importer [36], was also downregulated, again supporting that tryptophan concentrations were high. The repression of 13 phage-related genes (Table 2) upon deleting *tnaA *suggests a possible link in biofilm formation and phage-related genes as reported previously in *P. aeruginosa *[37]. The repression of seven motility genes in the *tnaA *mutant (Table 2) may be the reason the *tnaA *mutant does not increase biofilm formation despite low intracellular indole (Fig. 2). In addition, 22 translation and RNA genes were induced in the *tnaA *mutant, and 36 translation and RNA genes were also induced in the *trpE *mutant.

To corroborate further the microarray results (i.e., the genes identified as differentially-expressed in the microarrays should impact biofilm formation upon deletion), two independent cultures of the isogenic *E. coli *K-12 mutants *gadA *and *hdeA *were tested for altered biofilm formation using the 96-well crystal violet assay in LB and LB glu medium. Notably, the most significant changes occurred in LB glu medium where there were significant increases in biofilm (Fig. 4).

## Discussion

In this study we demonstrate that both indole and *N*-acylhomoserine lactones are signals in the formation of *E. coli *biofilms. Indole extracellular concentrations are significant in rich culture broths, and it appears indole negatively regulates *E. coli *biofilm formation by reducing motility (Fig. 6) and by influencing acid resistance. The differences in the architecture upon indole addition (Fig. 3A/3B) are most likely due to differences in cell motility since we have found differences in *E. coli *biofilm architecture between strains with different motility phenotypes [30], and the architecture of the strain without indole displayed similarities with high-motility strains while architecture with indole was similar to the architecture for low motility strains. Hence, just as one needs an accelerator as well as a brake to control an automobile, it appears *E. coli *cells control biofilm formation using an inducer (AI-2 [4]) as well as a repressor (indole). In contrast, indole stimulates biofilm formation for *P. aeruginosa*.

Along with stimulating pseudomonad biofilm formation, the two sets of dual-species biofilm results demonstrate that indole is an interspecies signal that can be manipulated by other bacteria (note there are 27 genera with a putative tryptophanase with a 30% identity to that of *E. coli*). This type of signal manipulation has been shown for AI-2 with *E. coli *and *V. harveyi *[38] as well as for homoserine lactone with *Erwinia carotovora *and *Bacillus thuringiensis *[39]; hence, competition and control for signals appears to be intense in biofilms. This competition extends beyond procaryotes as eucaryotes are well known for manipulating the quorum sensing signals of bacteria, too. For example, algae block bacterial biofilm formation by controlling both homoserine lactone and AI-2 signaling via furanones [40], and mammals (including humans) block homoserine lactone signaling via lactonase in sera [41]. Furthermore, it appears that the mechanism by which procaryotes (such as the pseudomonads in this study) manipulate the biofilm signal indole is through the relaxed substrate range of many dioxygenases and monooxygenases found in bacteria that bring about indole hydroxylation [27]; i.e., we propose that some of the oxygenases bacteria use for catabolism [42] have also evolved to regulate concentrations of the inter-species signal indole by removing it via precipitation: competitors that wish to remove indole simply oxidize it in one step to indigo which is insoluble and hence leaves the system.

The results presented here are also important in that they show clearly a connection between homoserine lactone signaling and biofilm formation in *E. coli*. This implies that *E. coli *uses the homoserine lactone signaling pathway to monitor at least indole and homoserine lactone compounds (this report) as well as uses the AI-2 signaling pathway [4,5] to control biofilms. Hence, although *E. coli *does not produce its own homoserine lactone via a signal synthase, it uses its homoserine lactone transcriptional regulator (SdiA) to monitor indole-producing strains as well as to monitor strains like *P. aeruginosa *(produces *N*-butyryl-*L*-homoserine lactone [32]), *P. syringae *(produces *N*-hexanoyl-*L*-homoserine lactone [33]), and *P. fluorescens *(produces *N*-octanoyl-*L*-homoserine lactone [33]). In addition, two groups have found SdiA induces the multi-drug efflux pump AcrAB of *E. coli *[18,21], and AcrAB has been hypothesized to control the efflux of quorum signals [18]. Given that we demonstrated TqsA of *E. coli *controls the efflux of the quorum signal AI-2 [5], and others demonstrated MexAB-OprM of *P. aeruginosa *controls the efflux of the quorum signal *N*-(3-oxododecanoyl)-*L*-homoserine lactone [43,44], it appears indole via its control of SdiA, may also control the efflux of quorum signals as well as control antibiotic resistance [7].

It needs to be ascertained whether indole itself binds to SdiA which has now been shown to bind four homoserine lactones [22,23]. The induction of *sdiA *upon addition of indole (ascertained through our microarrays), the decrease in *sdiA*-mediated transcription upon indole addition (shown via the β-galactosidase reporter of the *ftsQ2p *promoter), the lack of response in biofilm formation upon addition of homoserine lactones by the *sdiA *mutant, the lack of change in acid resistance upon indole addition by the *sdiA *mutant, and the fact that exogenous indole did not reduce the biofilm of the *sdiA *mutant, all suggest indole may bind SdiA. Furthermore, our results indicate that the extracellular factor first seen by García-Lara et al. [31] in an AI-2 minus background, which was shown to regulate the *ftsQ2p *cell division promoter via SdiA and to regulate *sdiA*, is indole. Significantly, indole and the AHLs have opposing effects on acid resistance and *ftsQ2p *transcription: indole reduced *ftsQ2p *transcription as well as reduced acid resistance but *N*-butyryl-*DL*-homoserine lactone increased *ftsQ2p *transcription in this study. Furthermore, *N*-decanoyl-*DL*-homoserine lactone is known to increase *ftsQ2p *transcription [45], and *N*-hexanoyl-*L*-homoserine lactone enhances acid resistance by inducing *gadA *more than 120-fold [23]. Hence, these results indicate different roles of indole and AHLs on acid-resistance via SdiA. Also, since indole controls biofilm formation through SdiA, these are some of the first results with SdiA showing a phenotypic change in *E. coli *K12 [46]. Since indole and *N*-butyryl-*DL*-homoserine lactone showed a marginal effect on plasmid-encoded *ftsQ2p *expression as previously reported [31,45], measurement of *ftsQ2p *expression from its natural position in the chromosome [19] would be required to corroborate our results.

We also propose that the DNA microarray analysis here provides insight into how the bacteria of the gastrointestinal (GI) tract may help to restrict access to pathogens and how it may regulate acid resistance, motility, and biofilm formation of non-pathogenic bacteria. An increase in extracellular indole in the GI tract by non-pathogenic bacteria may repress genes that encode regulators such as *gadX *(Table 2) and thereby reduce virulence in the duodenum since GadX activates virulence genes there [47]. Also, it appears that non-pathogenic *E. coli *may turn off acid resistance genes (*gad *and *hde *operons, Table 2) in the presence of indole in the weak basic gut flora since they are no longer needed, so indole serves to regulate acid resistance. Also, this increase in indole may serve to regulate motility in a complex manner (Table 2) and allow cells to form a biofilm. We speculate that the extracellular levels of indole increase only *after *the non-pathogenic biofilms have reached a certain critical thickness so that indole may decrease motility in the colonizing pathogenic bacteria and allow them to be more effectively removed from the gastrointestinal tract (indole is a negative chemoattractant for *E. coli *O157:H7, unpublished results). The pathogen *E. coli *O157:H7 may use indole as a signal since SdiA represses expression of the virulence factors EspD and intimin [20]. In addition, indole from bacteria is absorbed into body [48] so it easy to imagine that cells of the gastrointestinal tract may also manipulate indole levels to control bacteria.

Since we found extracellular concentration of indole decreases for K-12 cultures from 450 ± 70 μM to 15 ± 6 μM upon the addition of glucose to LB medium due to catabolite repression of *tnaA *[25], some of the results here and in our earlier reports [5,14,16,49] may need to be interpreted in light of changes in indole concentrations. For example, out of 93 isogenic knockout mutants identified with temporal biofilm microarrays, 70 mutants showed a more distinctive change in biofilm formation relative to the wild-type strain in LB glu medium compared to LB medium [49]. Here, the K-12 *trpE*, *tnaC*, *trpL*, *gadA*, and *hdeA *mutants had more significant biofilm changes in LB glu (Fig. 4, where indole concentrations are minimal) compared to LB.

**Figure 8:**
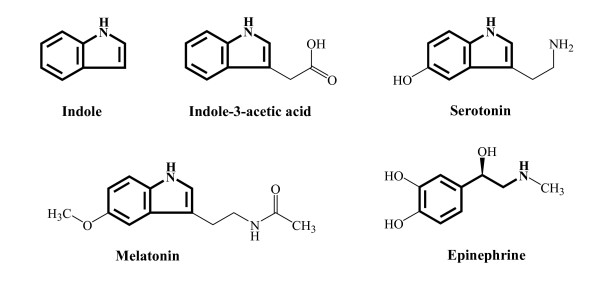
Indole, melatonin, serotonin, epinephrine, and indole-3-acetic acid. Indole motifs are in bold.

It is becoming clear that prokaryotes and eucaryotes signal not only themselves but also one another; for example, there appears to be crosstalk between *E. coli *O157:H7 and cells of the gastrointestinal tract through the hormones epinephrine and norepinephrine (catecholamines) [50]. Other hormones are also present in the gastrointestinal tract including melatonin [51] and serotonin [52]; both are neural hormones which maintain homeostasis and both reduce chlamydial infection [53]. In addition, plants use indole 3-acetic acid as their main hormone (for cell growth, division, tissue differentiation, and response to light and gravity), and bacteria interrupt this eucaryotic signaling by using indole-3-acetic acid as a source of carbon, nitrogen, and energy [54]. All five hormones, such as indole, indole-3-acetic acid, serotonin, melatonin, and epinephrine, have indole-like chemical structures (Fig. 8); hence, although it is highly speculative, it is intriguing to ponder whether indole was incorporated into the metabolism of eucaryotic hosts (plants and animals) and is the archetypal hormone.

## Conclusion

Indole is shown to clearly inhibit *E. coli *biofilm formation and DNA microarrays were used to determine that the mechanism of this inhibition is through SdiA. Indole is an interspecies biofilm signal as it is also shown here that it regulates pseudomonad biofilms, and it is shown that this signal can be manipulated by non-specific oxygenases in a dual-species biofilm to control the population of each bacterium. For the first time, it is also shown that both *E. coli *and pseudomonads respond to biofilm signals they do not synthesize.

## Methods

### Bacterial strains, materials and growth

The strains and plasmids used are listed in Table 3. LB [55] was used to pre-culture all the *E. coli *cells. LB glu was used for the single species biofilm experiments whereas LB was used for the dual species. To determine indole toxicity, *E. coli *K-12 was grown with 0 to 500 μM indole with dimethylformamide (DMF) added at 0.1% (v/v) to all samples. The *N*-acylhomoserine lactones were purchased from Aldrich (Allentown, PA).

**Table 3:** Strains and plasmids used. Amp^R^, Erm^R^, Tet^R^, and Km^R ^are ampicillin, erythromycin, tetracycline and kanamycin resistance, respectively.

**Strains and Plasmids**	**Genotype**	**Source**
**Strains**		
*E. coli *K-12 BW25113	*lacI*^q ^*rrnB*_*T14 *_Δ*lacZ*_WJ16 _hsdR514 Δ*araBA*-D_AH33 _Δ*rhaBAD*_LD78_	[67]
*E. coli *K-12 BW25113 Δ*trpE*	K-12 Δ*trpE::*Km^R^, defective in anthranilate synthase component I	[67]
*E. coli *K-12 BW25113 Δ*tnaC*	K-12 Δ*tnaC::*Km^R^, defective in the tryptophanase leader peptide	[67]
*E. coli *K-12 BW25113 Δ*trpL*	K-12 Δ*trpL::*Km^R^, defective in the *trp *operon leader peptide	[67]
*E. coli *K-12 BW25113 Δ*tnaA*	K-12 Δ*tnaA::*Km^R^, defective in tryptophan deaminase	[67]
*E. coli *K-12 BW25113 Δ*gadA*	K-12 Δ*gadA::*Km^R^, defective in the acid resistance gene glutamate decarboxylase A	[67]
*E. coli *K-12 BW25113 Δ*hdeA*	K-12 Δ*hdeA::*Km^R^, defective in acid resistance	[67]
*E. coli *K-12 BW25113 Δ*sdiA*	K-12 Δ*sdiA::*Km^R^, defective in sensing autoinducer-1	[67]
*E. coli *K-12 ATCC 25404	wild-type	ATCC
*E. coli *K-12 ATCC25404/pGEM-T RFP	Amp^R^, RFP^+^	this work
*E. coli *K-12 XL1-Blue/pCM18	*recA1, lac, endA1*, *gyrA96, thi, hsdR17*, supE44, *relA1/*F' *proAB*^+^, *lacI*^*q*^, *lacZ*ΔM13, Tn10 Tet^R^, Erm^R^, GFP^+^	this work
*E. coli *UT481/pCX39	Δ*lac-pro met pro zzz::*Tn*10 thy supD *r_K_^- ^m_K_^-^/*ftsQ2p::lacZ*, Amp^R^	[31]
*E. coli *WX2/pCX39	Δ*lac-pro met pro zzz::*Tn*10 thy supD *r_K_^- ^m_K_^- ^m_K_^- ^m_K_^-^/*ftsQ2p::lacZ*, Δ*sdiA::*Km^R^, Amp^R^	[31]
*E. coli *JM109	*recA1 supE44 endA1 hsdR17 gyrA96 relA1 thi *Δ(*lac-proAB*) F' *[traD36 proAB*^+^*lacI*^*q*^*lacZ*Δ*M15]*	[68]
*E. coli *TG1	*supE hsd*Δ5 *thi *Δ(*lac-proAB*) F' [*traD*36 *proAB*^+^*lacI*^*q *^*lacZ*ΔM15]	[55]
*P. aeruginosa *PAO1	wild-type	T. McDermott
*P. fluorescens *2–79	wild-type (NRRL B-15132) TOM^-^	[28]
*P. fluorescens *2-79TOM	*P. fluorescens *2–79 TOM^+^, Km^R^	[28]
*P. fluorescens *2-79TOM/pHKT3	TOM^+^, Km^R^, Tet^R ^RFP^+^	this work
*P. fluorescens *2–79/pHKT3	TOM^-^, Tet^R ^RFP^+^	this work

### Crystal violet biofilm assay

This assay was adapted [29]; *E. coli *was grown in polystyrene 96 well plates at 37°C for one day without shaking in LB glu medium except for the homoserine lactone experiment which was conducted at 30°C in LB medium. *P. aeruginosa *and *P. fluorescens *were cultured in LB at 30°C. Each data point was averaged from twelve replicate wells (six wells from two independent cultures) and the standard deviations were calculated. Plates were processed after 24 hours. The experiments were performed two or four times using independent cultures.

### Single-species biofilms in flow cells

The inocula and biofilm growth medium was LB glu supplemented with 300 μg erythromycin ml^-1 ^to maintain pCM18 [56] to retain the constitutive GFP vector for visualizing the biofilm; 50 μg kanamycin ml^-1 ^was used for the inocula of the *trpE *and *tnaC *mutants. The biofilm was formed at 37°C in a continuous flow cell and visualized with confocal microscopy as described previously [30]. The inocula were diluted to a turbidity of 0.05 at 600 nm and used to seed the flow cell for 2 h at 10 ml h^-1 ^before fresh LB glu erythromycin medium was added at the same flow rate. To study the effect of indole, indole in DMF was added at 500 μM upon inoculation and in the continuous feed; DMF was added as the negative control to the no-indole flow cell. The initial inoculum was 1.9 to 7.7 × 10^7 ^cells ml^-1^.

### Dual-species biofilms in flow cells

*E. coli *K-12 ATCC25404 harboring pGEM-T RFP, derived from pGEM-T (Promega, Madison, WI) and containing the constitutively expressed RFP, was cultured with either *P. fluorescens *2-79TOM (expresses constitutively toluene *o-*monooxygenase to convert indole to isoindigo [28]) or *P. fluorescens *2–79 as the negative control. TOM activity of *P. fluorescens *2-79TOM was determined to be 0.37 nmol/min mg protein using a naphthalene to naphthol assay based on HPLC [57]. LB medium with 100 μg ampicillin ml^-1 ^to maintain plasmid pGEM-T RFP was used to form biofilms at 32°C in the continuous flow cell (the two *P. fluorescens *strains were naturally-resistant to 100 μg ampicillin ml^-1^). RFP allowed visualization of the *E. coli *biofilm by excitation at 546 nm and emission at 590 nm. The experiment was performed in duplicate.

In a second dual-species biofilm system, both pseudomonads contained RFP via the broad-host-range plasmid pHKT3 [58] and *E. coli *K-12 XL1-Blue was used with GFP from pCM18 [56]; in this way, both bacteria were tagged with a flourophore. TOM was active in *P. fluorescens *2-79TOM/pHKT3 (RFP) (0.24 nmol/min mg protein). Also, the addition of TOM to *P. fluorescens/*pHKT3 did not reduce its growth rate. LB medium supplemented with 20 μg tetracycline ml^-1 ^(to maintain plasmid pHKT3 and to select for the *E. coli *host) was used to form biofilms at 32°C in the continuous flow cell (pCM18 was stable in *E. coli *without antibiotics but not in *P. fluorescens*). RFP allowed visualization of the *P. fluorescens *biofilm by exciting with a HeNe laser at 543 nm (emission 590 – 680 nm) and GFP allowed visualization of the *E. coli *biofilm by exciting with a Ar laser at 488 nm (emission 510 – 530 nm) using a TCS SP5 scanning confocal laser microscope with a 63x HCX PL FLUOTAR L dry objective with correction collar and numerical aperture of 0.7 (Leica Microsystems, Mannheim, Germany). Overnight cultures of both bacteria were diluted to a turbidity of 0.05 at 600 nm and used to inoculate the flow chamber for two hours at 10 ml h^-1 ^(roughly 2.6 × 10^7 ^cells ml^-1^). Fresh LB medium with 20 μg tetracycline ml^-1 ^was then introduced at the same flow rate and circulated for 7 days.

### Flow cell image analysis

Color confocal flow cell images were converted to gray scale [30], and biomass, substratum coverage, surface roughness, and mean thickness were determined using COMSTAT image-processing software [26] as described previously [30]. At each time point, from five to nine different positions were chosen for microscope analysis and 25 images were processed for each point. Values are means of data from the different positions at the same time point, and standard deviations were calculated based on these mean values for each position. Simulated three-dimensional images were obtained using IMARIS (BITplane, Zurich, Switzerland). Twenty-five pictures were processed for each three-dimensional image.

### Motility assay

LB overnight cultures were used to assay motility in plates containing 1% (w/v) tryptone and 0.25% (w/v) NaCl and 0.3% (w/v) agar [59]. The motility halos were measured at 8 h. When the effect of indole on motility was tested, indole (500 μM) dissolved in DMF was added to the motility agar. DMF (0.1%, v/v) was added as the negative control. Each experiment was performed two or four times using two independent cultures with each culture evaluated in triplicate. Also, it was confirmed that the motility with 0.1% (v/v) DMF was nearly identical to motility without DMF.

### Biofilm total RNA isolation for DNA microarrays

For all 3 sets of microarray experiments, 10 g glass wool (Corning Glass Works, Corning, N.Y.) were used to form biofilms [11] in 250 ml in 1 L Erlenmeyer shake flasks which were inoculated with overnight cultures diluted that were 1:100. For K-12 *yceP *with indole, 600 μM indole in 150 μL DMF or 150 μL DMF alone were added to cells grown in LB glu. For K-12 *tnaA *vs. K-12 wild-type, cells were grown in LB, and for K-12 *trpE *vs. K-12 wild-type, cells were grown in LB glu. The cells were shaken at 250 rpm and 37°C for 24 hours to form biofilms on the glass wool, and RNA was isolated from the biofilm as described previously [11].

### DNA microarrays

The *E. coli *Genechip antisense genome array (Affymetrix, P/N 900381) which contains probe sets for all 4290 open reading frames (ORF), rRNA, tRNA, and 1350 intergenic regions was used to study the differential gene expression profile for indole addition and for the *tnaA *and *trpE *mutants compared to the isogenic wild-type K-12 in a mature biofilm as described previously [4]. Hybridization was performed for 16 h and the total cell intensity was scaled automatically in the software to an average value of 500. The data were inspected for quality and analyzed according to the procedures described in Data Analysis Fundamentals which includes using premixed polyadenylated transcripts of the *B. subtilis *genes (*lys, phe, thr, dap*) at different concentrations. Also, as expected, there was insignificant *tnaA *and *trpE *mRNA signals in the biofilm of the *tnaA *and *trpE *mutants, and the completely-deleted *E. coli *K-12 BW25113 genes *araA *and *rhaA *showed insignificant mRNA levels. Genes were identified as differentially expressed if the expression ratio was greater than 2 and the change p-value is less than 0.05 since the standard deviations were 1.0 and 1.2 from duplicate experiments for indole, 1.4 for *tnaA*, and 1.2 for *trpE*. The gene functions were obtained from the National Center for Biotechnology Information database [60] and from the EcoCyc database [61,62]. The expression data for the biofilm samples have been deposited in the NCBI Gene Expression Omnibus [63] and are accessible through GEO Series accession number GSE4562 [64,65].

### Indole and β-galactosidase assays

Extracellular and intracellular indole concentrations from cells in LB medium were measured spectrophotometrically in duplicate as described previously [14] by modifying the protocol of Kawamura-Sato et al. [66]. Also, the spectrophotometric indole assay was corroborated with reverse-phase HPLC using a 100 × 4.6 mm Chromolith Performance RP-18e column (Merck KGaA, Darmstadt, Germany) and gradient elution with H_2_O-0.1% (v/v) formic acid and acetonitrile as the mobile phases at a flow rate of 1 ml min^-1 ^(65:35 for 0–5 min, 35:65 for 5–12 min, and 65:35 at 12 min). Under these conditions, the retention time for indole was 5.9 min, and the absorbance maximum was 271 nm.

*E. coli *UT481 harboring pCX39 (*ftsQ2p::lacZ*) and *E. coli *WX2 (*sdiA*-) harboring pCX39 (*ftsQ2p::lacZ*) [31] was grown at 30°C with 100 μg ampicillin ml^-1 ^from diluted overnight cultures to a turbidity of 1.0 or 2.0 at 600 nm. The β-galactosidase activities were calculated based on a protein concentration of 0.24 mg protein ml^-1 ^O.D_600_^-1 ^as reported previously [49].

### Acid resistance assay

This assay was adapted [35]. Overnight cultures grown for 19 h in LB were re-grown to mid-log phase in LB (turbidity at 600 nm of 1), and the culture was diluted 40-fold into phosphate-buffered saline (pH 7.2) or LB (pH 2.5) at 37°C. *E. coli *in LB (pH 2.5) was incubated at 37°C for 1 h without shaking. Indole was also added during the acid challenge to determine its effect. The percentage of cells surviving the acid treatment was calculated as the number of colony forming units (CFU) ml^-1 ^remaining after acid treatment divided by the initial CFU ml^-1 ^at time zero.

## Authors' contributions

JL designed research, performed experiments, analyzed the data, and helped draft the manuscript. AJ participated in the design of the study. TKW conceived the study, designed experiments, analyzed data, and wrote much of the manuscript. All authors read and approved the final manuscript.
